# A Remotely Delivered Progressive Walking Intervention for Adults With Persistent Symptoms of a Mild Traumatic Brain Injury: Feasibility and Exploration of Its Impact

**DOI:** 10.3389/fresc.2022.898804

**Published:** 2022-07-06

**Authors:** Christophe Alarie, Isabelle Gagnon, Elaine de Guise, Michelle McKerral, Marietta Kersalé, Béatrice van het Hoog, Bonnie Swaine

**Affiliations:** ^1^École de Réadaptation, Faculté de Médecine, Université de Montréal, Montréal, QC, Canada; ^2^Institut Universitaire sur la Réadaptation en Déficience Physique de Montréal (IURDPM), Montréal, QC, Canada; ^3^Centre de Recherche Interdisciplinaire en Réadaptation du Montréal Métropolitain (CRIR), Montréal, QC, Canada; ^4^School of Physical and Occupational Therapy, Faculty of Medicine and Health Sciences, McGill University, Montréal, QC, Canada; ^5^Trauma Center and Pediatric Emergency Medicine, Montreal Children's Hospital, McGill University Health Center and Research Institute of the McGill University Health Center, Montréal, QC, Canada; ^6^Département de Psychologie, Faculté des Arts et des Sciences, Université de Montréal, Montréal, QC, Canada

**Keywords:** walk, exercise, physical activity, mTBI, concussion, mixed-methods, mild traumatic brain injury, feasibility study

## Abstract

**Introduction:**

Persistent post-concussion symptoms following a mild traumatic brain injury (mTBI) can impact function and participation of adults. Physical activity is recommended to reduce symptoms and foster return to normal activities. Adults with a mTBI may have personal factors or experience accessibility issues restricting physical activity. Walking is a physical activity accessible to most that could be delivered remotely.

**Objectives:**

Determine the feasibility, safety, and acceptability of a remotely delivered progressive walking intervention designed for adults with persistent mTBI symptoms and explore its effects on health-related outcomes.

**Methodology:**

This feasibility study using a single-group pre-post mixed methods convergent parallel design was conducted remotely. Adults aged 18–65 years with a mTBI reporting persistent symptoms for ≥3 months were recruited. The 8-week remote progressive walking intervention aimed to increase the weekly number of steps walked by 40% based on a 1-week baseline measured by a Fitbit Inspire 2 activity monitor. Feasibility measures were about the intervention, its remote delivery, safety, and acceptability. Health-related outcomes were post-concussion symptoms, kinesiophobia, mood, sleep, fatigue, and quality of life. Semi-structured exit interviews were recorded and transcribed verbatim. Quantitative and qualitative data were analyzed separately, and results merged, compared, and contrasted. Descriptive statistics and paired samples *t*-tests were used. The qualitative analyses followed an iterative content analysis approach using reflexivity and triangulation of sources.

**Results:**

Twenty adults (16 women) aged 42.5 ± 11.51 years with persisting symptoms for 9.25 ± 6.43 months participated, adhered to 94.38% of sessions, completed the intervention, and found it to be feasible, safe and acceptable. Participants increased weekly total number of steps walked (change = 14,886 ± 18,283; *t* = 3.55, *p* = 0.002). Severity of post-concussion symptoms (change = −6.42 ± 10.69; *t* = −2.62, *p* = 0.018), kinesiophobia (change = −5 ± 6.86; *t* = 3.18, *p* = 0.005), anxiety (change = −1.53 ± 3.01; *t* = −2.21, *p* = 0.04), and fatigue (change = −10.21 ± 10.20; *t* = −4.37, *p* < 0.001) were reduced, whilst quality of life improved (change = 10.58 ± 13.35; *t* = 3.46, *p* = 0.003). Participants' perceptions corroborate most quantitative results; they felt improved self-efficacy about physical activity and provided five key recommendations.

**Discussion:**

This study demonstrates the feasibility, safety, and acceptability of the remote 8-week progressive walking intervention, a promising approach to reduce persisting symptoms, improve physical activity level health-related outcomes and quality of life of adults with persistent post-concussion symptoms following a mTBI.

## Introduction

Adults who sustain a mild traumatic brain injury (mTBI) can experience a range of physical, cognitive, and emotional post-concussion symptoms that persist and negatively impact their function and participation [e.g., work absenteeism, reduced physical activity (PA)] (1–4). Recent reviews and meta-analyses suggest that PA can help reduce persisting symptoms and fosters the return to normal activities (5–9). This evidence supports clinical practice guidelines and expert consensus on sport-related concussion that slow-to-recover adults should engage in PA as part of the management of their mTBI (10–12).

Among PA-based interventions delivered to adults with a mTBI, one of the most promoted approaches is symptom-limited low-to-moderate aerobic exercise performed five–seven times a week at an intensity representing 80% of the heart rate attained when symptoms are exacerbated during a graded exertion test, repeated every 2–3 weeks (13–16). However, there are several limitations associated with this approach. For instance, it requires regular testing of exercise intolerance, in-person supervision and precise monitoring of heart rate during exercise that may not be optimal for all adults with persisting symptoms (e.g., inactive, or aging adults). Indeed, this heterogenous population could experience several barriers to engage and follow this approach as they could be sedentary (17), fear-avoidant (18, 19) and may not have access to specialized equipment (e.g., treadmills, stationary bikes) or rehabilitation if living in a remote area or when it is limited due to sanitary restrictions (e.g., reduced service provision and increased waitlists).

An exercise as simple as walking is an aerobic-based PA accessible to most, that can be delivered remotely and has shown to improve perceived stress and mood of adults with mild, moderate, or severe TBIs living in the community (20). The home-based walking intervention studied by Bellon et al. (20) used a stepwise progressive approach using a pedometer to increase by 40% the total weekly steps walked after a 12-week intervention, an individualized progression that did not require symptom exacerbation to guide exercise progression. Although, this intervention incorporated telephone coaching, it required in-person testing before the intervention, and was not specifically designed for adults with a mTBI. Moreover, Bellon's study did not measure post-concussion symptoms and relevant mTBI health-related outcomes (e.g., anxiety, fatigue, sleep, quality of life) nor did it report on safety and participant acceptability.

In the context of the COVID-19 pandemic, we adapted Bellon's approach and designed a remote progressive walking intervention specifically for adults with persisting post-concussion symptoms of a mTBI. The aim of this study was to determine the feasibility, safety, and acceptability of this 8-week telehealth intervention, and to explore its effects on health-related outcomes.

## Materials and Methods

This study was conducted entirely remotely and followed a single-group pre-post intervention using a mixed method convergent parallel design (21). In other words, we simultaneously collected different qualitative and quantitative data before, during and after the intervention, analyzed both sets of data separately, and combined and compared (e.g., triangulated) the results from these data sources to draw conclusions. The study was approved by the Center for Interdisciplinary Research in Rehabilitation of Greater Montreal (CRIR) Research Ethic Board (#CRIR-1516-1118).

### Participants

**Figure 1:**
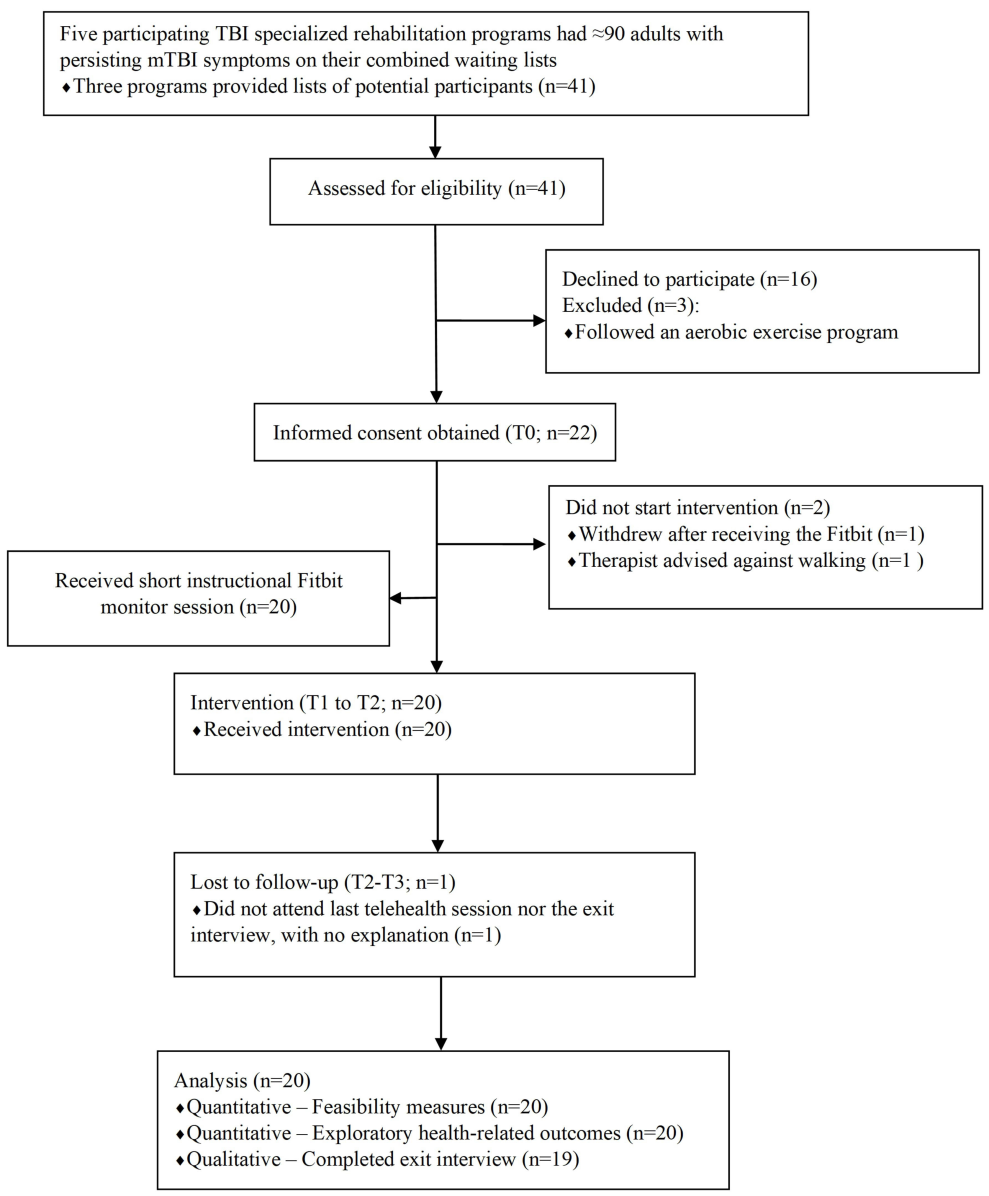
CONSORT diagram of the flow of participants in the study.

Eligible participants were adults aged 18–65 years old reporting persistent post-concussion symptoms following a mTBI sustained ≥3 months and for ≤3 years before and whose names were on waitlists to receive outpatient rehabilitation at one of five public healthcare TBI specialized programs in Montréal and surrounding area, an urban region in Québec, Canada. To be on the waitlist of a specialized TBI rehabilitation program, participants had a diagnosis of a mTBI and were referred to the program by a physician. Participants had to speak English or French and have access to the internet, a web camera and a microphone on a computer, a smartphone, or a tablet with Bluetooth^©^ able to support Zoom software (Zoom Video Communications Inc., USA provided by the Université de Montréal). Potential participants were excluded from the study if they (1) reported not feeling healthy enough to walk daily [informed by the Physical Activity Readiness Questionnaire (PAR-Q)], (2) reported an injury or a disease such as a sprain, a fracture or testing positive for COVID-19 during the study, or (3) were already following an aerobic exercise program. At all times during their participation in the study, participants could receive services from their doctor, allied health professionals (e.g., physiotherapist, occupational therapist, kinesiologist) and alternative health professionals (e.g., acupuncturists, massage therapists) working inside or outside the specialized program as long as no PA intervention was provided. Non-probabilistic convenience sampling was used until 20 participants were consecutively enrolled and initiated the intervention, a sample consistent with other feasibility studies of PA interventions for youth and children with mTBI (13, 22). Participants were recruited from May to October 2021 by persons independent from the research team from each specialized program who made initial contact calls to individuals on waitlists, briefly introducing the study and inquiring about interest in participating. Once potential participants demonstrated interest, they were contacted by telephone by the first author (CA) to confirm eligibility. An email describing the intervention including the consent form and a secured Zoom URL of the first scheduled session was then sent to each participant (T0). This session consisted of obtaining consent and demographic information through screen sharing, in addition to scheduling the period to obtain baseline walking data and planning the telehealth intervention sessions. Figure 1 reports the flow of participants in the study.

### Telehealth Progressive Walking Intervention

The 8-week walking intervention was remotely delivered and consisted of weekly telehealth sessions during which research assistants met with participants to individualize their walking progression and offer motivational support specific to individuals with a mTBI. The intervention aimed to increase the weekly number of steps walked, up to an additional 40% of the steps walked during the 1-week baseline measured with a Fitbit Inspire 2 PA monitor (Fitbit LLC, USA). This progression was inspired by the progression proposed by Bellon et al. (20). Specifically, participants were encouraged to follow a progression rule to increase the number of steps walked per week by 5% of the number of steps walked during baseline, and this was adapted weekly.

During weekly telehealth sessions, research assistants and participants worked collaboratively to establish individualized walking goals for the week informed by the potential obstacles and facilitators to walking perceived by the participants for each upcoming week. Participants could modulate the frequency, duration, distance, and speed of their walks to achieve their goals. To promote attainment of their walking goals, motivational strategies were provided (23). They were also educated about PA and mTBI (e.g., potential health benefits for mTBI symptoms, exercise intolerance principles), and encouraged to attain desired behaviors through feedback, and helped to identify strategies to achieve walking goals (e.g., goal setting, action planning). Participants were asked to record the frequency, the durations of their walks, and their ratings of perceived effort after voluntary walks in an electronic Word software-based (Microsoft Word, Microsoft, USA) walking log to support self-monitoring of their progress. Specific details about the intervention are provided in Supplementary Material I according to the Consensus on Exercise Reporting Template checklist (24).

For each participant, the intervention included 10 individual telehealth sessions supervised by trained research assistants, one session to initiate baseline measurement (T0), two sessions for questionnaire administration, pre and post intervention (T1 and T2), and seven weekly-scheduled sessions for data collection, weekly goal setting and motivational support. Not considered as part of the intervention, participants attended a 20-min instructional session about the use of their Fitbit (between T0 and T1), and an exit interview (T3) within one week after finishing the intervention.

The research assistants were the first author (CA), a doctoral student with over five years of clinical experience, and three students at the master's or undergraduate level in occupational therapy or athletic therapy. Supervised by a senior researcher (BS), the doctoral student provided 20 h of training to the other students to ensure standard delivery of the walking intervention, administration of the measurement tools, data collection and data entry. To ensure intervention and protocol fidelity, pilot testing of the intervention and assessment procedures (e.g., consent, wearing Fitbit, questionnaire completion, exit interview) was conducted with members of the research team. Pilot data were not included in the study.

### Procedure

Participant demographic data (see Table 1) were collected at T0 and included questions about self-reported level of PA (e.g., number of days performing more than 30 min of PA at an intensity that made them breathe harder than usual, the number of minutes of PA per week, and self-rated level of fitness). Questionnaires were administrated at the beginning (T1) and end of the 8-week intervention (T2) followed by an exit interview (T3). Demographic information and questionnaire responses were collected using Zoom screen sharing and annotation tools. The total number of mTBI sustained in one's life was self-reported. Within four days of obtaining consent, participants received in the mail their Fitbit Inspire 2 activity monitor and were invited via email to attend the Fitbit instructional session (between T0 and T1) during which they were instructed how to download, install, and perform initial set-up of the Fitbit application. They were asked to wear the watch on their non-dominant wrist and go about their days as usual for one week to complete the baseline (T0–T1). A piece of masking tape was placed on the watch face to impede participants from viewing their daily number of steps during this initial week and they were instructed to remove the tape only once the intervention began (T1). For the study duration, participants were instructed to always wear the watch but could remove it when bathing or while it charged. A checklist was also provided with the mail package to remind participants about these instructions.

**Table 1:** Demographic characteristics of participants receiving the telehealth progressive walking intervention (*n* = 20).

**Variable**	**Participants (*n* = 20)**
Age (years), mean ± SD, *range*	42.5 ± 11.4, *20–59*
Woman, *n* (%)	16 (80)
Above high school diploma, *n* (%)	9 (45)
Post-concussion symptoms at T0, (RPQ total score)	33.90 ± 15
Time since injury (month), mean ± SD, *range*	9.25 ± 6.4, *3–29*
**Prior mTBI**, ***n*** **(%)**	
0	17 (85)
1	2 (10)
2	1 (5)
**Concussion mechanism**, ***n*** **(%)**	
Sport or physical activity-related	3 (15)
Fall	4 (20)
Motor vehicle accident	4 (20)
Physical violence	2 (10)
Struck by an object	4 (20)
Unspecified work-related	3 (15)
**Employment status**, ***n*** **(%)**	
Full-time	3 (15)
Student	2 (10)
Progressive return-to-work	2 (10)
Sick leave	12 (60)
Retired	1 (5)
**Self-reported premorbid condition**, ***n*** **(%)**	16 (80)
ADD/ADHD	6 (30)
Depression	5 (25)
Anxiety	3 (15)
Learning disability	1 (5)
Migraine	1 (5)
Days ≥30 min of moderate PA, mean ± SD	1.6 ± 2.1
Min of PA per week, mean ± SD	69.25 ± 81.3
**Self-rated level of fitness**	
Much less fit or somewhat less fit	14 (70)
Equally as fit or somewhat more fit	6 (30)

*SD, standard deviation; RPQ, Rivermead Post-Concussion Symptom Questionnaire; mTBI, mild traumatic brain injury; ADD/ADHD, attentional deficit disorder/attentional deficit hyperactive disorder; PA, physical activity*.

Each week, participants received a personal secured Zoom link by email connecting them to a 30-min one-on-one telehealth virtual meeting with their assigned research assistant. A typical telehealth session unfolded as follows: Participants shared their walking log and synchronized their Fitbit Inspire 2 with the Fitbit application, allowing the research assistant to access steps data. The research assistant and participant then discussed the weekly number of steps, goal attainment, identified actual and potential barriers and facilitators (e.g., fatigue, busy schedule, motivation) and planned new goals for the upcoming week. The time of the next appointment was confirmed and an email containing an updated walking log, new weekly goals and a Zoom link was sent to the participants by email. Weekly reminders were sent by email 24 h before each appointment. At the end of each telehealth session, the research assistant recorded in a journal if participants received health services, if they reported adverse events, if any technological issues occurred during the session and general comments participants shared about the intervention. If an adverse event was reported either during a telehealth session, with a phone call or an email, research assistants were instructed to tell the participant to seek medical attention, as needed. If medical attention was required, the plan was to maintain contact (by telephone or email) with the participant until he or she felt the consequences related to the adverse event were under control. Depending on the severity of the adverse event, a decision to continue or withdraw from the study would be made by the research team.

### Exit Interview

An exit interview (T3) with each participant was conducted remotely by CA. Using an interview guide based on the acceptability questionnaire developed for this study (see below), participants were asked five open-ended questions about their overall satisfaction with the intervention delivery and the telehealth experience. Interviews were recorded using built-in Zoom recording software and transcribed verbatim.

### Feasibility and Outcome Measures

Feasibility measures and feasibility level criteria, set *a priori* when possible and aligned with prior research for determining feasibility of PA interventions (13, 25) included: Drop-out rate, measured as three or more missed telehealth sessions or a participant declining to continue the intervention (acceptable drop-out rate set at ≤20%); Adherence to the telehealth sessions (target attendance set at ≥80% of the telehealth sessions for all participants); Number of modifications to scheduled telehealth sessions; Number of adaptations of telehealth sessions; Type of device used to support the telehealth platform; Number of internet connection, video and audio issues; Number of Zoom platform issues; Number of Fitbit monitor issues and; Walking log usage, determined as the number of logs completed and shared with the research team (≥80% of the walking logs). Safety was measured as the number of minor (e.g., fall, minor injury, perceived exacerbation of symptoms due to walking) and major adverse events (e.g., subsequent mTBI, an emergency department visit) resulting from walking. Finally, the acceptability of the intervention with respect to its different characteristics (e.g., duration, frequency of meetings, format, research assistants' expertise and know-how), telehealth setting (e.g., Zoom connection quality), Fitbit activity monitor, perceived impacts of the intervention were assessed using a 17-item 5-point scale questionnaire (1 = strongly disagree, 2 = disagree, 3 = neither agree nor disagree, 4 = agree, 5 = strongly agree) designed for this study (see Supplementary Material II).

### Exploratory Health-Related Outcomes

The exploratory health-related outcomes included:

- Steps walked over seven days (i.e., number of steps walked per 7 × 24-h periods), measured with a wrist-worn Fitbit Inspire 2 watch, a validated PA monitoring technology in adult population with TBI (26). Additionally, self-reported weekly number of walks, walk duration, and the rating of perceived effort (RPE) using the Borg rating of perceived effort scale, a 0–10-point scale commonly used for individuals with a mTBI (13, 27–29), were recorded by participants in their electronic walking log.
- Post-concussion symptoms, assessed with the Rivermead Post-Concussion Symptom Questionnaire (RPQ), a self-reported validated and reliable 16-item questionnaire using a 5-point scale (total score range = 0–64) (30). Three sub-scores can be calculated, namely cognitive, somatic, and affective symptoms. A higher score indicates a greater number or intensity of symptoms.
- Kinesiophobia, measured by the Tampa Kinesiophobia Scale (TSK-13) a self-reported questionnaire validated for adults with a mTBI including 13 items measured on a 4-point scale (total score range = 13–52) (31). Two sub-scores can be calculated: activity avoidance and somatic focus (i.e., fear of pain and (re)injury). A higher score indicates greater fear.
- Mood, measured with the Hospital Anxiety and Depression Scale (HADS), a self-reported questionnaire validated for adults with a mTBI including 14 items measures using a 4-point scale (total score range = 0–42) (32, 33). Two sub-scores can be calculated representing: depression and anxiety. A higher score represents a higher level of symptoms.
- Sleep quality over the past month, assessed using the Pittsburgh Sleep Quality Index (PSQI), a self-reported questionnaire validated for individuals with a TBI containing 19 items measuring seven domains of sleep on a 4-point scale (total score range = 0–21) (34). A higher score represents poorer perceived sleep quality.
- Fatigue, measured with the Multidimensional Fatigue Inventory (MFI) a self-reported questionnaire validated for adults with a TBI containing 20 items measured using 5-point scale (total score range = 20–100) (35, 36). Five sub-scores are calculated: general, physical, mental, and emotional fatigue and vigor. A higher score indicates a greater level of fatigue.
- Health-related quality of life, measured with the Quality of Life after Brain Injury (QOLIBRI), a self-report questionnaire validated for individuals with a mTBI comprised of 37 items measured using a 5-point scale (total score reported as a percentage) (37). Six sub-scores can be calculated: cognitive, self-perception, life, relational, emotional, and physical satisfaction. A higher score represents a better quality of life.

### Analyses

This mixed-method study used different methodological techniques to gather quantitative and qualitative data. The analyses of each type of data were performed separately and results from these data sources were triangulated.

Descriptive statistics were performed for demographic, feasibility, safety, acceptability, and health-related outcome data. Visual inspection was performed for distribution and completeness of feasibility and health-related outcomes. Satisfaction questionnaire scores were transformed into percentages. Due to the exploratory nature of the study, paired sample Student's *t*-tests were used to analyze changes in exploratory health-related outcomes (e.g., steps walked, post-concussion symptoms, kinesiophobia, mood, sleep, fatigue, health-related quality of life) before (T1) and after the 8-week intervention (T2). Sensitivity analyses based on the Wilcoxon signed-ranked test were performed and conclusions were coherent with those of the Student's *t-*tests, except for the mental fatigue sub score of the MFI standardized questionnaire (MFI-Mental). Moreover, using paired sample Student's *t-*test enables the reporting of Cohen's *d* effect sizes (38). Quantitative analyses were performed using SAS software (SAS 9.4, SAS Institute, Inc., USA).

Qualitative analyses of exit interviews and journals kept by each research assistant were performed principally by CA, MK and BH and supervised by BS using Nvivo software (Nvivo 1.6, QSR International Inc., USA). The iterative analysis process followed Miles et al. (39) analysis approach. A coding dictionary derived from the interview guide was produced to inform the first cycle of coding. The coding dictionary evolved as transcripts were coded independently and verified by a second analyst. Suggested changes or adjustments were resolved through discussion between analysts and the first author CA. A second cycle of coding determined broader categories. To enhance trustworthiness, triangulation of the broader categories with data recorded in the research assistants' journals provided enriched understanding of participants' experiences but did not modify the overarching categories. Annotations (*jottings)*, reflexive notes and analytical memos were documented thorough the analysis. A search for convergent, divergent, and contradictory information within categories was undertaken and reported (39).

The results of the quantitative and qualitative analysis related to each other were merged, compared, and contrasted to provide an overall interpretation of the feasibility, safety, acceptability, and impacts on health-related outcomes (21). Perspectives of participants including converging, divergent and contrasting results are reported following related quantitative results.

## Results

Demographic characteristics of the participants are in Table 1. Twenty French-speaking adults on waitlists of three outpatient rehabilitation programs participated in the 8-week intervention. They each completed the intervention, but one participant only provided the number of steps recorded during week 8 by the Fitbit and did not complete the self-reported questionnaires (T2) or the exit interview (T3). At the beginning of the intervention (T1), all participants reported having symptoms of fatigue, 95% reported feeling slow and being sensitive to noise, and 90% reported having headaches, light sensitivity and difficulty to concentrate and remember. Nineteen participants reported having received health services during the intervention by either a medical doctor (*n* = 3), an allied health professional (*n* = 16), or an alternative health professional (*n* = 5). Following the qualitative content analysis, perspectives of participants were grouped in five broader categories: feasibility, safety, acceptability, impacts on health-related outcomes, and recommendations to improve the walking intervention.

### Feasibility

All participants completed the intervention (0 drop-outs). Adherence to the telehealth sessions was 94.38%, 25 sessions were rescheduled, and 10 no-shows were recorded. Thirty-six sessions (18.94 %) diverged from the intervention protocol and needed adaptations (e.g., used telephone, session longer than 45 min, vacation). Twenty-four computer-related technological issues, 33 Zoom issues, and 53 Fitbit activity monitor issues were recorded but these issues were dealt with during the session and did not prevent completion of any session. Visual inspection of the number of steps data revealed that one participant did not wear his watch for three days during week 8 (T2) and was therefore removed from the analysis of the weekly steps. Table 2 provides more details about the feasibility measures.

**Table 2:** Feasibility measures of the telehealth progressive walking intervention (*n* = 20).

**Variable**	**Frequency (%)**
**Adherence to telehealth sessions (*****n*** **= 200)**	190 (95)
Schedule modifications	37 (18.5)
Rescheduled telehealth sessions	26 (13)
No-shows without reasons	25 (12.5)
No-show rescheduled	15 (7.5)
Telehealth session adaptation recorded over the 190 sessions^*^	36 (18.94)
Phone line uses instead of telehealth platform	27 (14.21)
Telehealth sessions ≥45 min	8 (4)
Adaptations due to vacation	3 (2)
Sessions needing to be split into two	2 (1)
**Device supporting telehealth platform used over the 190 sessions** ^**a**^	
Computer	112
Smartphone	62
Tablet	22
**Technological issues recorded over the 190 sessions**	24 (12.63)
Internet connection	12 (6.59)
Poor audio quality	9 (4.74)
Poor video quality	3 (1.58)
**Zoom platform issues recorded over the 190 sessions**	33 (17.37)
Device incompatibility	22 (11.58)
Connectivity	11 (5.79)
**Fitbit activity monitor issues recorded over the 190 sessions**	53
Participants reported to have forgotten to wear Fitbit	14 (26.42)^b^
Participants perceived lack of Fitbit validity	20 (37.74)^b^
Participants reported charging issues	15 (28.30)^b^
Participants reported difficulty synchronizing with Fitbit software	4 (7.55)^b^

* *Excluding the no-shows*.

a *Sometimes, participants concurrently used two devices*.

b *Percentage calculated on the total frequency of Fitbit activity monitor issues recorded*.

Walking logs were completed by participants following 69.44% of the sessions. Self-reported information on walking logs was however sparse making statistical analysis difficult: 14 participants provided information to compare walk frequencies between week 1 and 8, eight participants for the duration of walks, and seven participants about their rating of perceived effort. Perceptions of participants converged with quantitative feasibility results providing insights about the intervention and issues experienced. Participants found the telehealth progressive walking intervention feasible and felt the quality of the telehealth sessions was good; it was simple and quick to communicate with the research team by email. Technical assistance offered by the research team was good and instructions provided throughout the intervention were clear even when technological issues occurred. Using the Fitbit activity monitor and its online application were considered feasible and helpful for tracking their PA even if issues or dissatisfaction were expressed about the perceived reliability of the Fitbit's step counts measurement, the relatively frequent charging of the watch and its lack of screen brightness outside. The walking log was considered useful to track symptoms and walking progression, but some people experienced difficulties completing the walking log because of their visual symptoms or the electronic format.

### Safety

We recorded 21 adverse events during the study, 19 of which were exacerbation of post-concussion symptoms after a walk and two musculoskeletal (e.g., knee pain, and calf cramp). No event required medical attention, and all were resolved without complications. Experience of participants converged with the overall safety of the intervention regarding the low frequency of musculoskeletal minor adverse events, contextualized symptom exacerbation, and perceived potential hazards. Indeed, participants considered the walking a safe activity and appreciated that the individualized progression and adapted weekly goals did not put them at risk of overexerting themselves or increasing their symptoms. Some participants felt their safety was at risk (e.g., risk of fall) in the presence of symptoms of dizziness and light sensitivity when walking on uneven terrain and when they walked in an area considered unsafe, or during inclement weather (e.g., storm, heatwave).

### Acceptability

Overall acceptability was high. The percentage of participants who responded 4 (agree) or 5 (strongly agree) to all items about the intervention itself was 79%, the remote delivery was 68%, the instrumentation and tools used was 74%, and the perceived health benefits was 79%. All would recommend the intervention to persons with a mTBI. Converging with these results, during the exit interview participants shared their appreciation of the intervention, its telehealth format, its 8-week duration, its weekly frequency, and the relatively short duration of telehealth sessions (about 30 min.). Although some participants found the experience more time consuming than initially expected (e.g., data collection, time to complete walking logs, number of telehealth sessions), participating in the research was a positive experience for everyone; they found the data collected helped them better understand persisting post-concussion symptoms. Research assistants were considered knowledgeable motivators, flexible with scheduling and able to adapt intervention progression according to individual needs. Participants reported wanting the telehealth approach to be embedded in the public healthcare system and would have liked to have received a similar walking program earlier after their injury. They reported the pandemic enhanced their readiness to participate in a telehealth intervention and found themselves familiar/comfortable with the technology used (i.e., Zoom, email exchanges, Fitbit, and its online application). Although participants generally appreciated the intervention in the format given, five areas of improvement emerging from the interviews were found. Table 3 reports main recommendations and significant excerpts from participants.

**Table 3:** Participants' recommendations and excerpts to improve the remotely delivered interventions (*n* = 19).

**Recommendations**	**Excerpts (quotes)**
**1. The walking intervention should be a multimodal intervention** • Make intervention multimodal that includes other types of PA other than walking and including dedicated educational modules on persisting symptom management, sleep, nutrition, and PA in general for individuals with mTBI • Add an interactive component with participants to experience and learn from each other	“Well, you know, I'm someone who likes to move around, and who likes to try a lot of things. It would have been more diversified. (…) You have four or five days a week that you walk, then you have two days that you can either do a little jogging, or a brisk walk, or whatever. You know, as the program goes on, the [activities of the days] might change.” –* Line, 38 years old, initially feeling less active than her peers*
**2. The length of the walking intervention should be prolonged** • Extend intervention at least another 4 weeks with the goals of better consolidating lifestyle habits related to PA, prolonging access to supervision to maintain motivation toward PA, and integrating/experiencing new PA	“I would have taken a little more. I would have taken 10 or 12 [weeks]. Yes. To see if... I was still able to walk more steps. At the beginning it was a challenge (…). Then after that it was really okay! Now, I get out of the house and then it's… wow! I guess I could have done more, but it would have taken ten to twelve [weeks].” –* Catherine, 47 years old, initially feeling less active than her peers*
**3. Reinforce personalization of the intervention** • Adjust intervention to better meet individual needs. Increase frequency of weekly meetings or formulate walking goals more challenging than simply increasing 40% of steps per week • Better tailor measures for tracking PA to individual's needs, preferences, and comfort with technology; for example, in the walking log, record more details about the daily presence of symptoms or record activities besides walking	“I would have found it interesting if the final objectives were personalized for each person. That the person discusses with you to find a final goal that would better suit him. Because mine, I found that it was a little too low according to my walking abilities. That made it so that I was sure that I was going to reach the final goal but like, way above what it was. So, I would have found it interesting, either for the next ones, if the goals were personalized for each person, so that it would be more representative of their abilities and then of their personal goals.” –* Eva, 20 years old, initially feeling more active than her peers*
**4. Better preparation of participants for the intervention** • Make more explicit the required participation, and expectations of the intervention including better explanation about using the walking diary, the Fitbit watch and its online application and the questionnaires • Send participants questionnaires in advance to facilitate data collection/evaluation and add more reminders to charge the watch	“When I had to connect it [the Fitbit], with my account on my cell phone, I had a paper in the box with the code, and the password and the username. Well, I admit, it's really stupid, but personally I looked for it. I didn't come across this paper right away. Then I was like “*Ok, but how do I plug it in?*” So, the paper that was in the box, maybe put it outside the box. Maybe...mention it more clearly. I don't know, something like that. (…) Just taking the watch, logging in, and then the steps to... that little bit was less clear.” – *Sara, 44 years old, initially feeling less active than her peers*
**5. Optimizing measurement validity** • Ensure measures of the number of steps are representative of PA during a typical week. Indeed, some reported that the PA level during the baseline week was either too low or too high	“In my case, I think it came into play [the validity of the Fitbit]. Because it happened that the first week was the beginning of my vacation, and I was more in couch potato mode! But, still I was moving around, so my baseline is not so low either, but maybe not quite the representation of my life at one hundred percent.” –* Diane, 40 years old, initially feeling more active than her peers*

*PA, physical activity; mTBI, mild traumatic brain injury*.

### Exploratory Health-Related Outcomes

Participants significantly (*p* < 0.01) increased their weekly total number of steps and their average daily number of steps after the intervention, during the weekday and weekends (see Table 4) and reported the intervention impacted positively their participation. In fact, not only did they report an increased number and duration of their walks, they also spent more time in work-related and leisure activities. For example, some participants reported starting to cycle, swim or paddle board and having improved their ability to work on the computer because of screen habituation during the one-on-one telehealth session. Often, participants included their friends and their family during their walks or other PA resulting in strengthened relationships with their friends and family.

**Table 4:** Descriptive statistics, paired sample *t*-test and effect size of steps walked, walk frequencies and duration, and perceived effort of the telehealth progressive walking intervention (*n* = 20).

**Walk-related**	**Baseline**	**Post-Intervention**	**Change [CI]**	***t*** **-statistic**	***p*** **-value**	**Cohen's *d***
**outcome**	**(T0-T1; *n* = 20)**	**(Week 8-T2; *n* = 19)**	
Total weekly steps, mean ± SD	57,881.60 ± 21,228.19	74,854.95 ± 27,743.62	14,886.21 ± 18,282.73 [6,074.21 to 23,698.21]	3.549	**0.002**	0.687
Daily steps, mean ± SD	8,268.81 ± 3,032.57	10,831.62 ± 4,158.11	2,264.65 ± 2,868.97 [881.85 to 3,647.45]	3.441	**0.003**	0.707
Daily weekday steps, mean ± SD	8,544.9 ± 3,678.24	10,940.01 ± 4,420.02	2,087.69 ± 3,150.65 [569.13 to 3,606.26]	2.888	**0.010**	0.589
Daily weekend steps, mean ± SD	7,578.60 ± 4,089.73	10,560.63 ± 4,157.71	2,707.05 ± 4,049.51 [755.25 to 4,658.85]	2.914	**0.009**	0.723
Weekly walk frequencies, mean ± SD^*^	4.88 ± 2 (*n* = 16)	4.63 ± 2.42 (*n* = 16)	−0.29 ± 2.7 [−1.27 to 1.85] (*n* = 14)	−0.396	0.699	0.113
Weekly walk duration, mean ± SD^*^	22.45 ± 8.51 (*n* =10)	38.43 ± 26.79 (*n* = 16)	10.97 ± 16.17 [−2.56 to 24.49] (*n* = 8)	1.918	0.097	0.804
RPE, mean ± SD^*^	2.97 ± 2.94 (*n* = 10)	3.27 ± 1.66 (*n* = 14)	−0.86 ± 2.67 [−3.34 to 1.61] (*n* = 7)	0.953	0.758	0.126

*Bold represents statistical significance*.

^*^n varies since not all data were recorded in personal logs.

*SD, standard deviation; RPE, rating of perceived effort; CI, confidence interval*.

Paired sample *t*-tests indicated that post-concussion symptoms (RPQ-Total), kinesiophobia (TSK-13-Total), anxiety (HADS-Anxiety), and fatigue (MFI-Total) reduced post-intervention (*p*-values for all four measures = <0.05), while health-related quality of life (QOLIBRI-Total) improved (*p* = 0.003). Initial and final scores for each questionnaire and their associated subscales, confidence intervals, *t*-statistics, *p*-values, and Cohen's *d*, are provided in Table 5. Similarly, participants qualitatively reported a reduction in their fatigue, their cognitive, emotional, and physical symptoms of mTBI, and that their overall physical condition improved over the course of the intervention (i.e., they had stronger legs and improved cardiorespiratory function). Converging with the quantitative results, they felt the intervention improved some cognitive abilities (e.g., sustained attention), their overall mood, sleep quality (e.g., reduced stress, irritability), and reduced their pain. Some participants reported the intervention helped them lose weight.

**Table 5:** Descriptive statistics, paired sample *t*-test and effect size of post-concussion symptoms, kinesiophobia, mood, sleep, fatigue, and health-related quality of life exploratory health-related outcomes (*n* = 20).

	**Pre-intervention**	**Post-intervention**	**Change [CI]**	***t*** **-statistic**	***p*** **-value**	**Cohen's *d***
	**(T1; *n* = 20)**	**(T2; *n* = 19)**				
**Symptoms**						
RPQ-total (0–64)	33.9 ± 15.56	26.58 ± 15.19	−6.42 ± 10.69 [−11.58 to −1.27]	−2.617	**0.018**	0.476
RPQ-somatic	17.4 ± 7.08	14 ± 7.99	−3 ± 7.99 [−6.17 to 0.17]	−1.989	0.062	0.450
RPQ-emotional	7.1 ± 5.66	5.95 ± 4.4	−1 ± 3.35 [−2.61 to 0.61]	−1.301	0.210	0.227
RPQ-cognitive	7.5 ± 2.96	5.58 ± 3.15	−1.68 ± 2.47 [−2.88 to −0.49]	−2.968	**0.008**	0.628
**Kinesiophobia**						
TSK-13-total (13–52)	36.55 ± 7.51	31.89 ± 8.8	−5 ± 6.86 [−8.31 to −1.69]	−3.175	**0.005**	0.570
TSK-somatic focus	14.6 ± 3.79	12.47 ± 3.78	−1.95 ± 3.19 [−3.48 to −0.41]	−2.662	**0.016**	0.563
TSK-activity avoidance	21.95 ± 4.77	19.42 ± 5.42	−3.05 ± 4.18 [−5.07 to −1.04]	−3.181	**0.005**	0.541
**Mood**						
HADS-total (0–42)	19.90 ± 9.59	17 ± 9.66	−2.79 ± 6.21 [−5.78 to 0.20]	−1.959	0.066	0.301
HADS-anxiety	11.15 ± 4.70	9.47 ± 4.54	−1.53 ± 3.01 [−2.98 to −0.08]	−2.212	**0.040**	0.364
HADS-depression	8.75 ± 5.17	7.53 ± 5.56	−1.26 ± 3.75 [−3.07 to 0.55]	−1.466	0.160	0.227
**Sleep**						
PSQI-total (0–21)	10.5 ± 4.24	8.63 ± 4.21	−1.42 ± 3.19 [−2.96 to 0.11]	−1.944	0.067	0.443
**Fatigue**						
MFI-total (20–100)	48.65 ± 9.02	38.16 ± 11.21	−10.21 ± 10.20 [−15.12 to −5.30]	−4.365	**<0.001**	1.031
MFI-general	16.85 ± 2.58	13.53 ± 3.84	−3.16 ± 3.22 [−4.71 to −1.61]	−4.276	**<0.001**	1.015
MFI-physical	14.95 ± 3.05	12.16 ± 4.18	−2.84 ± 3.27 [−4.42 to −1.27]	−3.787	**<0.001**	0.763
MFI-mental	15.3 ± 3.51	13.42 ± 4.4	−1.68 ± 3.15 [−3.20 to −0.17]	−2.333	**0.032**	0.472
MFI-emotional	13.25 ± 3.71	9.53 ± 4.9	−3.68 ± 4.57 [−5.89 to −1.48]	−3.513	**0.003**	0.856
MFI-vigor	11.7 ± 3.37	10.47 ± 4.75	−1.16 ± 3.52 [−2.85 to 0.54]	−1.435	0.168	0.299
**Health-related quality of life**						
QOLIBRI-total (0–100)	55.54 ± 17.16	67.11 ± 17.94	10.58 ± 13.35 [4.15 to 17.02]	3.456	**0.003**	0.660
QOLIBRI-cognition	18.1 ± 6.23	22.16 ± 7	3.68 ± 4.70 [1.42 to 5.95]	3.414	0.296	0.613
QOLIBRI-self perception	17.2 ± 6.47	22.68 ± 7.48	5.26 ± 7.29 [1.75 to 8.78]	3.148	**0.006**	0.784
QOLIBRI-life satisfaction	19.9 ± 6.05	24.68 ± 7.35	4.37 ± 5.98 [1.48to 7.25]	3.182	**0.005**	0.710
QOLIBRI-relation	19.75 ± 6.15	22.89 ± 5.25	2.84 ± 4.66 [0.60to 5.09]	2.659	**0.039**	0.549
QOLIBRI-emotion	14.45 ± 5.72	16.16 ± 5.08	1.63 ± 3.48 [−0.05 to 3.31]	2.041	0.056	0.316
QOLIBRI-physical	13.35 ± 4.66	15.58 ± 4.9	1.79 ± 3.43 [0.14to 3.44]	2.277	**0.002**	0.466

*Bold represents statistical significance*.

*RPQ, Rivermead Post-Concussion Symptom Questionnaire; TSK-13, Tampa Kinesiophobia Scale; HADS, Hospital Anxiety and Depression Scale; PSQI, Pittsburgh Sleep Quality Inventory; MFI, Multidimensional Fatigue Inventory; QOLIBRI, Quality of Life after Brain Injury; CI, Confidence Interval*.

Additionally, the participants reported improved self-efficacy related to persisting post-concussion symptoms management and PA. They learned more about mTBI, its associated symptoms, the general health benefits of PA and how PA could specifically help improve their mTBI. They reported an increased sense that PA could be beneficial in managing their persisting symptoms and felt that, sometimes, a single walk made them feel better. Helped by the research assistants, participants felt supported to engage in a reflective process about themselves, their physical capacities, symptoms status, PA goals, and self-management skills of symptoms (e.g., energy level, pain). They felt they had a better balance in their lives because the intervention provided structure to their day, contributing to improved personal time management. They felt overall positive impacts on motivation, self-esteem, self-confidence, enjoyment, and well-being due to the walking intervention.

## Discussion

This paper reports on a remotely delivered 8-week progressive walking intervention for adults with persisting symptoms of a mTBI. The results showed the intervention was feasible, safe, and acceptable by adults waiting to receive outpatient rehabilitation. It thus contributes to emerging telehealth approaches for use with individuals with a mTBI (40, 41).

The results suggest the intervention meets the needs of adults with a mTBI because participants were heavily engaged in the intervention. Indeed, they reported being satisfied with all its components and shared they would have liked to have received it earlier in their recovery process. Moreover, there were no drop-outs, and the adherence was similar to that found for other PA interventions (25, 42), and higher than most interventions delivered to individuals with a mTBI (28, 43–46). Most studies about PA intervention for individuals with a mTBI do not even report adherence (7).

Why the participants were so appreciative of the intervention remains somewhat unclear. We hypothesize that the type of PA offered (i.e., low intensity PA in a progressive manner) was probably very appropriate for the participants who were found to have PA levels below those recommended by the Canadian Physical Activity Guidelines (e.g., 150 min of moderate to vigorous aerobic PA per week) (47). Following the intervention, their weekly steps walked, and the daily average steps walked approached numbers similar to those of healthy individuals (48). Walking, or moving around, is essential to participation in real life situations in community, social and civic life, and appears to be non-threatening PA for adults who are slow to recover from a mTBI. However, it remains unclear if walking will remain an activity of choice outside of a pandemic context.

Perhaps the participants appreciated the intervention because the research assistants provided motivational support using behavioral change techniques improving participants' motivation and self-efficacy to be more active (23). Contained in weekly telehealth sessions of <30 min, we provided information about PA and their mTBI, helped with goal setting and action planning, provided feedback on behavior, thus supporting self-monitoring and habit formation to help participants walk more. Little is known about self-efficacy concerning PA among adults with a concussion, however evidence suggests that self-efficacy of self-management behaviors influences participation, life satisfaction and health-related quality of life in persons with newly acquired brain injury, including TBI (49). Moreover, Gagnon et al. (50), found that self-efficacy about PA in children was reduced after a concussion (50). In our recent scoping review of PA interventions for individuals with a mTBI, only about 22% of studies (*n* = 8/35) on interventions for individuals with a mTBI reported using motivational support in conjunction with a PA intervention (7). Our study suggests the importance of providing motivational strategies when delivering a PA intervention to adults with a mTBI.

It is conceivable that participants appreciated the intervention simply because they felt better. In fact, they reported reduced persisting post-concussion symptoms, several improved health-related outcomes, and their quality of life. Interestingly, using a mixed methods design allowed identification of some divergent results between self-reported questionnaires and participant experiences. For example, participants subjectively reported improved sleep following the intervention, but changes in the PSQI questionnaire total scores did not differ significantly. Also, some reported pain reduction, while others felt they improved their physical fitness and even lost weight. Since walking is an aerobic PA performed at an intensity typically lower than most existing approaches offered to individuals with a mTBI (7), this suggests that the mechanisms underlying improvements in persisting symptoms may not only be related to physiological effects driven by more intense aerobic exercises (e.g., jogging, biking, running), but also about doing exercise itself, an activity often considered pleasurable and associated with leisure-time activity. Given the lack of a control group in this feasibility study, we cannot presume the improvement in symptom status and health-related outcomes are solely related to the PA intervention.

In terms of safety, although participants reported symptom exacerbations after some walking, they reported symptoms resolved by themselves, and no one wanted to drop-out or stop walking because of this. This suggests the walking intervention is no less safe than other PA interventions for individuals with a mTBI, which often report temporary increase in post-concussion symptoms because of exercising (13, 41, 44, 51).

### Clinical Implications

Although the intervention was deemed beneficial, feasible and safe, there were some challenges. First, the rescheduling of telehealth sessions was resource intensive. Because most of these participants were not working or at school at the time, we were often able to reschedule appointments during weekdays, but sometimes scheduling was only possible in the evening and weekends. This could be a challenge when delivering this intervention in either a public or private healthcare setting. Also, the Fitbit watches required troubleshooting by the research assistants throughout the intervention. Before delivering this intervention, research assistants were trained and became familiar with the most common functions on the Fitbit and with its application. This training proved essential as it ensured research assistants could answer most questions and provide technical support. Since participants were not convinced the watches were able to accurately record their PA, extra time was required to teach participants about how the Fitbit detects steps (how it works). Third, although the walking logs were completed almost 70% of the time, their completeness was greatly variable. This made using the walking logs difficult during the intervention to inform walking progression and made it challenging to perform statistical analysis. As some participants reported the walking log to be helpful for self-monitoring progress, and because research assistants needed a measurement of steps to inform future progression, in future research, we recommend dropping the participant Word-based walking log and using only the Fitbit application as the walking log. Indeed, the application has a built-in detailed log that automatically records the daily step count, the frequency, duration, and type of PA, and has the options for recording extra notes by the participants. For example, with the Fitbit, they could note symptom status before and after a walk, or other PA. Additionally, ecological momentary assessments such as sending text messages, or emails containing questions about their symptom status or their perceived effort at planned or random times, could be used to reduce recall bias and improve ecological validity (52). The walking log may be helpful clinically to support self-management of PA and symptoms.

Despite these challenges, this intervention appears to be a promising relatively low-cost approach to promote participation, reduce post-concussion symptoms and improve health-related outcomes among adults with a mTBI. However, we do not know if the changes in health-related outcomes measured with the standardized questionnaires met the minimal clinical important difference because of the lack of consensus about this metric for the instruments when used with adults with a mTBI. Nonetheless, this intervention could be helpful to gradually increase PA level of individuals less active than most, potentially deconditioned and who may want to avoid PA. This telehealth approach can be used to deliver a PA intervention to individuals that cannot access gym equipment, live in remote areas or as a starting point for individuals that experience kinesiophobia (e.g., fear-avoidant). The suggestions gathered during the interviews with our participants about including other modalities, lengthening the intervention, and reinforcing the personalization of the intervention could help further tailor the intervention to needs of adults with a mTBI. Considerations of these recommendations when adapting or implementing this intervention as part of outpatient rehabilitation services is warranted.

### Limitation and Future Directions

The results of this study should be interpreted with caution due to some limitations. First, although the Fitbit technology is valid to measures steps while walking (26), it is somewhat challenging to use step counts when it is worn during a 24-h period. Participants often shared their concerns about the accuracy of the watch to measure steps while they performed other activities reinforcing that the Fitbit technology may have accuracy issues in “free-living settings” (53). Second, the study is at risk for selection bias. Participants were consecutively recruited in a non-probabilistic manner from different waitlists and about 50% of those contacted declined to participate. Study participants may have been more motivated than most and thus not be representative of the heterogeneous adult population of persons with mTBI. Although the sample comprised mostly of woman (80%) differs from those included in a scoping review of studies on PA for individuals with a mTBI (7), these results could contribute to better understanding the perspectives of women with a mTBI participating in a PA intervention. The small sample and the wide range in time since injury (i.e., heterogeneity) limit the generalizability of the findings. The range of time since injury, however, represents the clinical reality of TBI specialized programs involved in our study and highlights that a telehealth PA intervention is a promising approach for individuals with different times since injury. Third, neither participants or research assistants were blinded to the intervention which may have influenced reporting of symptoms, and outcome measurements. To minimize this bias, the research assistants were trained, and they repeatedly informed the participants that the main objective of the study was to determine the feasibility and that criticism and transparency about the intervention was important. Fourth, we cannot draw conclusions about the intervention's effectiveness on health-related outcomes due to the lack of a control group and because of the health services most participants received either from their doctors, their allied or alternative health professionals. No participant received another PA intervention. Furthermore, we cannot conclude whether improvements reported by participants are maintained over time because of the lack of follow-up measures. A future randomized controlled trial including longer term follow-up measures could determine the extent to which this telehealth PA intervention influences post-concussion symptoms, health-related outcomes, and quality of life.

## Conclusion

A telehealth progressive walking intervention for adults with persisting symptoms of a concussion is feasible, safe, and acceptable. Participants of the intervention were highly satisfied and provided recommendations to improve the walking intervention. An 8-week walking intervention may be appropriate to integrate into the management of adults with an mTBI. More controlled research with lower risks of bias is warranted in the future to determine the effectiveness of this intervention to increase PA and to evaluate its effects on health-related outcomes.

## Data Availability Statement

The raw data supporting the conclusions of this article will be made available by the authors, without undue reservation.

## Ethics Statement

This study involving human participants was reviewed and approved by the Center for Interdisciplinary Research in Rehabilitation of Greater Montreal (CRIR) Research Ethics Board. Written informed consent for participation was not required for this study in accordance with the national legislation and the institutional requirements.

## Author Contributions

CA, BS, IG, EG, and MM conceived, designed the study, and obtained funding. BS oversaw data collection of both quantitative and qualitative analyses. CA coordinated data collection, performed the analysis, drafted the manuscript, and submitted the final version once validated by co-authors. MK and BH supported data collection and helped with quantitative data preparation and qualitative analysis. BS, IG, EG, MM, MK, and BH provided critical revisions of the manuscript and approved the final version. All authors reviewed and approved the final manuscript submitted.

## Funding

This research and the open access publication fees has been supported by the Fonds de Recherche du Québec en Santé (FRQS). CA received a bursary of Excellence from the École de Réadaptation of the Université de Montréal.

## Conflict of Interest

The authors declare that the research was conducted in the absence of any commercial or financial relationships that could be construed as a potential conflict of interest.

## Publisher's Note

All claims expressed in this article are solely those of the authors and do not necessarily represent those of their affiliated organizations, or those of the publisher, the editors and the reviewers. Any product that may be evaluated in this article, or claim that may be made by its manufacturer, is not guaranteed or endorsed by the publisher.
