# Supplementary material for: A Remotely Delivered Progressive Walking Intervention for Adults With Persistent Symptoms of a Mild Traumatic Brain Injury: Feasibility and Exploration of Its Impact
Source: Front Rehabil Sci. 2022 Jul 6;3:898804. doi: 10.3389/fresc.2022.898804 (PMC9397951; doi:10.3389/fresc.2022.898804)
Supplement: Supplementary file 1 [file Table_1.docx]

**Supplementary Material I:** Characteristics of the remote progressive walking intervention using the Consensus on Exercise Reporting Template (CERT) checklist.

| Section | Intervention Characteristic |
| --- | --- |
| **What: Materials** |  |
| 1. Description of the type of exercise equipment | Requirements for participants:   - Computer, intelligent phone or tablet with a webcam, microphone, and Bluetooth. - Email address - Shoes for outdoor walking   Requirements for service provider:   - Fitbit Inspire 2 watch activity monitor   - Online Fitbit application account - Zoom licence - Computer |
| **Who: Provider** |  |
| 2. Description of the qualifications, expertise and/or training | Research assistants*:   - 1 Kinesiologist, doctoral student - 1 Occupational therapist, master’s degree student - 1 Occupational therapist, undergraduate student - 1 Athletic therapist, undergraduate student   **All research assistants received ongoing supervision by the senior scientist (BS) and participated in a 20-hour training period where they learned about the research protocol, questionnaires administration, communication with research participants, Fitbit handling, data collection and quality control, intervention and interviewing.* |
| **How: Delivery** |  |
| 3. Describe if exercises are performed individually or in a group | The walking intervention was delivered individually.   - Participant, however, was free to walk individually or with peers (e.g., family, friends). - Participant had no contact with other participants. |
| 4. Describe whether exercises are supervised or unsupervised | The progressive walking is supervised:   - The participants performed their walks unsupervised. - Weekly telehealth sessions were performed during which research assistants supervised progression and adjusted steps goals if needed. |
| 5. Description of how adherence to exercise is measured and reported | Adherence was based on attendance to the 10 telehealth sessions required to initiate and complete the progressive walking intervention:   - 1 initial session to explain how the Fitbit monitor works and start the baseline. - 1 session for questionnaire administration and launch of the 8-week intervention. - 7 weekly telehealth sessions - 1 session for outcome measurement and perceptions about satisfaction of intervention at the end of the 8-week intervention |
| 6. Detailed description of motivational strategies | Motivational strategies* included were:   - Goal setting, action planning, review goals, discrepancy between current behaviour and goals, behavioural contracts. - Feedback on behaviour, self-monitoring of behaviour and outcomes, feedback on the outcome of behaviour. - Social support. - Shaping knowledge about physical activity and mTBI, behavioural experiments. - Habit formation, graded tasks.   **We used Michie et al., 2013 behavioral change technique taxonomy to structure included motivational strategy.* |
| 7a. Description of the decision rule(s) for determining exercise progression | Participants aimed to increase their total number of weekly steps by 40% more than the total steps walked during the week of baseline at the end of the 8-week intervention. Research assistants calculated the progression for each participant and suggested each week an increase of 5% of the number of baseline weekly steps. |
| 7b. Description of how the exercise program was progressed | Participants and their research assistant worked together weekly to set and achieve weekly walking goals*:   - If the participant did not achieve his or her weekly objective, a new objective suitable for the following week was determined with the participant. - If the participant achieved his or her weekly objective of 5%, a new objective for next week (i.e., an increase of 5% of the step count of the baseline weekly step was suggested. - Participant attaining > 40% of the baseline weekly steps, no higher goal was suggested.   * *Participants often used not only steps, but minutes of walking, distances, frequencies to help them set their own goals.* |
| 8. Description of each exercise to enable replication | Walking was the only type of physical activity. |
| 9. Description of any home programme component | The progressive walking intervention is a home program and includes no other exercise component than walking. |
| 10. Description of any non-exercise components | Participants had to wear their Fitbit monitor at all times except when they bathed or charged their watch. |
| 11. Describe the type and number of adverse events that occurred | Minor adverse events*:   - A fall: An unexpected event leading to the participant falling during walking. (n=0) - A new injury: A musculoskeletal injury that occurs while the person was walking and is judged to be minor by the assessor (e.g., ankle sprain, knee sprain). (n=2) - Sustained increase in post-concussion symptoms: A sudden and sustained increase in post-concussion symptoms that occurs during or following a period of walking or PA. (n=19)   Major adverse event:   - A new injury: A concussion or musculoskeletal injury that occurs when the individual is performing a more challenging period of walking or PA and is deemed majors by the research team (e.g., a fracture). (n=0) - An emergency room visit: An injury or event resulting from a period of walking or other PA that requires an emergency room visit (n=0) |
| **Where: Location** |  |
| 12. Describe the setting in which the exercises are performed | Some individuals walked inside (treadmill) and most outside (e.g., parks, streets, woody area). |
| 13. Description of the exercise intervention | A normal telehealth session unfolded as follows (less than 30 minutes):   - At the beginning of each session participants were asked to send in their walking log. - The participant synchronized his Fitbit monitor with the Fitbit application, allowing the research assistant to access steps data. - Research assistants and participants discussed the number of steps, if they reached their goals, what facilitated or obstructed walking, planned new goals for the upcoming week and envisaged potential facilitators and obstacles to newly form walking goals. - Research assistant confirmed the participant next appointment, sent by email an updated walking log with the new weekly goals. |
| **Tailoring: what, how** |  |
| 14a. Describe whether the exercises are generic or tailored | The type of exercise is walking, and it is generic. |
| 14b. Description of how exercises are tailored to the individual | N/A |
| 15. Describe the decision rule for determining the starting level | The number of steps to walk set for the first week of the intervention is based on an increase of 5% of a participant total number of steps walked during a 1-week baseline.   - For example, a participant walking a total of 33,131 steps during baseline would have to walk a total 34,788 steps during the first week (5% = 1656 steps). |
| **How well planned: actual** |  |
| 16a. Describe how adherence or fidelity to the intervention protocol is assessed/measured | This feasibility study reports on several feasibility measures:   - Adherence to the intervention - Telehealth outcomes - Safety - Acceptability - Exploratory outcomes |
| 16b. Describe the extent to which the intervention was delivered as planned | Results about the feasibility can inform the extent to which the intervention was planned (please see the results section). |
