# Supplementary material for: A Remotely Delivered Progressive Walking Intervention for Adults With Persistent Symptoms of a Mild Traumatic Brain Injury: Feasibility and Exploration of Its Impact
Source: Front Rehabil Sci. 2022 Jul 6;3:898804. doi: 10.3389/fresc.2022.898804 (PMC9397951; doi:10.3389/fresc.2022.898804)
Supplement: Supplementary file 2 [file Table_2.docx]

**Supplementary Material II:** Progressive Walking Intervention in a Telehealth Setting for Adults Experiencing Persistent Symptoms of a mTBI satisfaction questionnaire

**Satisfaction Questionnaire**

**Please indicate your level of agreement with the following statements:**

|  | Strongly agree=5 | Agree=4 | Neither agree or disagree=3 | Disagree=2 | Strongly disagree=1 |
| --- | --- | --- | --- | --- | --- |
| **Intervention** |  |  |  |  |  |
| 1. The progressive walking intervention is overall satisfying. |  |  |  |  |  |
| 2. The duration of the progressive walking intervention is adequate. |  |  |  |  |  |
| 3. The duration of the meetings with the evaluator is adequate. |  |  |  |  |  |
| 4. The weekly step progression is adequate. |  |  |  |  |  |
| 5. The progressive walking intervention is safe. |  |  |  |  |  |
| 6. The technical support given by the evaluator during the progressive walking intervention is sufficient. |  |  |  |  |  |
| 7. The motivational support given by the evaluator during the progressive walking intervention is sufficient. |  |  |  |  |  |
| **Telehealth** |  |  |  |  |  |
| 8. It is easy for me to connect to the online Zoom meetings with the evaluator. |  |  |  |  |  |
| 9. The audio and video quality during the meetings is good. |  |  |  |  |  |
| 10. The internet connection during the meetings with the evaluator is good. |  |  |  |  |  |
| **Equipment** |  |  |  |  |  |
| 11. The physical activity monitor (Fitbit watch) is easy to use. |  |  |  |  |  |
| 12. The physical activity monitor is reliable to record data (number of steps). |  |  |  |  |  |
| **Impact of Intervention** |  |  |  |  |  |
| 13. I increased my level of physical activity because of the progressive walking intervention. |  |  |  |  |  |
| 14. I reached my walking goals during the intervention. |  |  |  |  |  |
| 15. I experienced negative impacts from the intervention. |  |  |  |  |  |
| 16. I experienced positive impacts from the intervention. |  |  |  |  |  |
| **Overall satisfaction** |  |  |  |  |  |
| 17. I would recommend this intervention to another adult experiencing persistent symptoms following a mTBI. |  |  |  |  |  |
